# Fetal growth velocity references from a Chinese population–based fetal growth study

**DOI:** 10.1186/s12884-021-04149-x

**Published:** 2021-10-09

**Authors:** Tianchen Wu, Xiaoli Gong, Yangyu Zhao, Lizhen Zhang, Yiping You, Hongwei Wei, Xifang Zuo, Ying Zhou, Xinli Xing, Zhaoyan Meng, Qi Lv, Zhaodong Liu, Jian Zhang, Liyan Hu, Junnan Li, Li Li, Chulin Chen, Chunyan Liu, Guoqiang Sun, Aiju Liu, Jingsi Chen, Yuan Lv, Xiaoli Wang, Yuan Wei

**Affiliations:** 1 Department of Maternal and Child Health, School of Public Health, Peking University, Beijing, 100191 China; 2 Department of Obstetrics and Gynecology, National Clinical Research Center for Obstetrics and Gynecology, Peking University Third Hospital, Beijing, 100191 China; 3 Department of Obstetrics and Gynecology, Maternal and Child Health Hospital of Qinhuangdao, Qinhuangdao, 066000 China; 4 Department of Obstetrics, Maternal and Child Health Hospital of Hunan, Changsha, 410007 China; 5 Department of Obstetrics, Maternal and Child Health Hospital of Guangxi Zhuang Autonomous Region, Nanning, 530000 China; 6 Department of Obstetrics, Tongzhou Maternal and Child Health Hospital of Beijing, Beijing, 101100 China; 7 Department of Obstetrics and Gynecology, the First Affiliated Hospital of Xiamen University, Xiamen, 361003 China; 8 Maternal and Child Health Hospital of Dongchangfu District, Liaocheng, 252004 China; 9 Department of Obstetrics, Gansu Maternal and Child Health Hospital, Lanzhou, 730050 China; 10 Department of Obstetrics and Gynecology, Changchun Obstetrics–Gynecology Hospital, Changchun, 130042 China; 11 Fujian Maternity and Child Health Hospital, Affiliated Hospital of Fujian Medical University, Fu Zhou, 350122 China; 12 Department of Function, Maternal and Child Health Hospital of Shijiazhuang, Shijiazhuang, 050051 China; 13 Children’s Hospital of Shanxi, Women Health Center of Shanxi, Taiyuan, 030013 China; 14 Department of Obstetrics, the First Affiliated Hospital of Chongqing Medical University, Chongqing, 400010 China; 15 Department of Obstetrics, Zhengzhou Central Hospital, Zhengzhou, 450007 China; 16 Department of Obstetrics, Maternal and Child Health Hospital of Changzhi, Changzhi, 046011 China; 17 Department of Obstetrics, Maternity and Infant Hospital of Shenyang, Shenyang, 110014 China; 18 Department of Obstetrics, Hubei Maternal and Child Health Hospital, Wuhan, 430070 China; 19 Department of Obstetrics, Inner Mongolia Maternal and Child Health Hospital, Hohhot, 010060 China; 20 Department of Prenatal Diagnosis, the Third Affiliated Hospital of Guangzhou Medical University, Guangzhou, 510150 China; 21 Shengjing Hospital Affiliated to China Medical University, Shenyang, 117004 China

**Keywords:** Fetal growth, Fetal growth velocity, Estimated fetal weight, Longitudinal study

## Abstract

**Background:**

Fetal growth velocity standards have yet to be established for the Chinese population. This study aimed to establish such standards suitable for the Chinese population.

**Methods:**

We performed a multicenter, population–based longitudinal cohort study including 9075 low–risk singleton pregnant women. Data were collected from the clinical records of 24 hospitals in 18 provinces of China. Demographic characteristics, reproductive history, fetal ultrasound measurements, and perinatal outcome data were collected. The fetal ultrasound measurements included biparietal diameter (BPD), abdominal circumference (AC), head circumference (HC), and femur diaphysis length (FDL). We used linear mixed models with cubic splines to model the trajectory of four ultrasound parameters and estimate fetal weight. Fetal growth velocity was determined by calculating the first derivative of fetal size curves. We also used logistic regression to estimate the association between fetal growth velocities in the bottom 10th percentile and adverse perinatal outcomes.

**Results:**

Fetal growth velocity was not consistent over time or among individuals. The estimated fetal weight (EFW) steadily increased beginning at 12 gestational weeks and peaked at 35 gestational weeks. The maximum velocity was 211.71 g/week, and there was a steady decrease in velocity from 35 to 40 gestational weeks. The four ultrasound measurements increased in the early second trimester; BPD and HC peaked at 13 gestational weeks, AC at 14 gestational weeks, and FDL at 15 gestational weeks. BPD and HC also increased from 19 to 24 and 19 to 21 gestational weeks, respectively. EFW velocity in the bottom 10th percentile indicated higher risks of neonatal complications (odds ratio [OR] = 2.23, 95% confidence interval [CI]: 1.79–2.78) and preterm birth < 37 weeks (OR = 3.68, 95% CI: 2.64–5.14). Sensitivity analyses showed that EFW velocity in the bottom 10th percentile was significantly associated with more adverse pregnancy outcomes for appropriate–for–gestational age neonates.

**Conclusions:**

We established fetal growth velocity curves for the Chinese population based on real–world clinical data. Our findings demonstrated that Chinese fetal growth patterns are somewhat different from those of other populations. Fetal growth velocity could provide more information to understand the risk of adverse perinatal outcomes, especially for appropriate–for–gestational age neonates.

**Supplementary Information:**

The online version contains supplementary material available at 10.1186/s12884-021-04149-x.

## Introduction

Intrauterine growth marks the starting point of a 1000-day early life growth period. The quality of this growth can profoundly affect the likelihood of a child fulfilling their developmental potential [1, 2], and is closely related to health and disease in adults [3–5]. Fetal ultrasound measurements during pregnancy are the main indicators of the quality of intrauterine growth. Comparison with fetal size curves determines whether the fetus has a size appropriate for gestational age [6]. Ultrasound measurements below the 10th percentile of the fetal size curve denote small–for–gestational age (SGA) status; they are important diagnostic criteria for fetal growth restriction (FGR) [7–9], which increases the risk of adverse perinatal outcomes. Several fetal size curves have been developed for clinical practice [10–12]. Choosing a chart that is appropriate for the genetic background and living environment of the population to which it is applied could improve the diagnostic power of SGA and FGR.

Traditional fetal size curves can assess fetal size at a specific time point, but provide little insight into dynamic changes occurring during the growth process. Fetal growth velocity, defined as the growth per unit time (e.g., g/week), can provide more insight into the growth process during a given period. A retrospective study of 4,285 singleton pregnancies showed that 74% of antepartum fetal deaths were not SGA at the time of the last ultrasound examination [13]. Compared to traditional fetal size curves, fetal growth velocity improved the sensitivity of predictions of antepartum fetal death (26.1 vs. 56.5%) [13]. Although several fetal growth velocity charts have been published [14–19], there are none specifically intended for the Chinese population. This is the first study to develop a fetal growth velocity chart for the Chinese population.

Data were obtained from the Chinese Fetal Growth Study, a multi–center cohort study involving 24 hospitals in 18 provinces in China, which aimed to establish a fetal growth chart suitable for the Chinese population. The objective of the present study was to develop a fetal growth velocity chart for estimating fetal weight, biparietal diameter (BPD), abdominal circumference (AC), head circumference (HC), and femur diaphysis length (FDL) in the Chinese population. To facilitate clinical application, we devised a model to determine whether the fetal growth velocity between any two gestational weeks was below a given percentile on the velocity chart. Furthermore, we explored the association between fetal growth velocities in the bottom 10th percentile and adverse outcomes.

## Methods

### Study design and participants

The Chinese fetal growth study was a multicenter, population–based cohort study. Singleton pregnant women who delivered between September 1 and October 31, 2019 were recruited from 24 hospitals in 18 provinces (Table S1). We only included low–risk pregnant women in our study, excluding those with complications or other conditions. The exclusion criteria of present study were: (1) abnormal prenatal diagnosis (including Edward’s syndrome, Down’s Syndrome, Turner’s syndrome, intrauterine infection); (2) hemoglobin < 110 g/l during the first trimester; (3) hyperthyroidism/hypothyroidism; (4) infant deformity; (5) gestational associated hypertension (including gestational hypertension, chronic hypertension, and pre–eclampsia/eclampsia), gestational diabetes mellitus, receiving assisted reproduction; (6) diabetes, autoimmune disease, hypertension or other non–communicable diseases before pregnancy; (7) previous pregnancy complicated with pre–eclampsia/eclampsia, or HELLP syndrome, infant deformity, preterm birth or birth weight < 2500 g or > 4500 g; (8) smoking or drinking within 3 months of pregnancy or the first trimester; (9) histories of exposure to toxic, harmful, or radioactive materials; (10) long–term medication history (except conventional folic acid, calcium, vitamins, or iron). The recruitment and exclusion procedures were conducted by three physicians in each hospital; two of the physicians independently determined whether a participant met the exclusion criteria, with any disagreements being resolved by the third physician. All physicians were trained to ensure that they could apply the exclusion criteria accurately. This study was approved by the Peking University Third Hospital Medical Ethics Committee (approved number: 2021 No. 336-02).

### Data collection

We designed a standardized data collection form and established an online data acquisition system. All data were obtained from the medical records of the pregnant women, including demographic characteristics, reproductive history, ultrasound biometric measurements, and perinatal outcomes. Two medical staff in each hospital were trained in the entry of data into the electronic data system. All records were reviewed by our research team and returned missing values and outliers to the corresponding partner hospital for reverification.

### Ultrasound measurements

Ultrasound measurements were conducted in accordance with the Prenatal Ultrasound Guide (2012) [20]. All participants underwent at least three ultrasound examination to measure biometric parameter, including BPD, AC, HC, and FDL, between 12 gestational weeks and delivery. Gestational age was calculated according to the last menstrual period with a regular cycle of 21–35 days, as confirmed by early ultrasound. If the time of the last menstrual period was unclear, the gestational week was determined by ultrasound examination. The confirmation of gestational age is conducted by measuring the crown–rump length in the first ultrasound examination (during 11 to 13^+ 6^ gestational weeks), when crown–rump length longer than 84 mm, head circumference is measured to confirm gestational age [20, 21]. Each parameter was measured twice, and the average value was calculated. All measurements were obtained from the ultrasonic images with the highest magnification. The original values of all measurements, and the original ultrasonic images, were retained for random quality control spot checks, so that outliers could be traced.

### Adverse perinatal outcome

Adverse pregnancy outcome was defined as a composite outcome, including SGA, neonatal complications, admission to the neonatal intensive care unit (NICU), premature rupture of membranes (PROM), and preterm birth < 37 gestational weeks. SGA was defined as birth weight in the bottom 10th percentile using the newly published gender–specific Chinese fetal birth weight standards [22]. Using the same gender–specific fetal birth weight standards, large–for–gestational age (LGA) was defined as birth weight in the 90th percentile, and appropriate–for–gestational age (AGA) as birth weight between the 10th and 90th percentiles. Neonatal complications included birth defects, jaundice, intrauterine infection, respiratory apnea syndrome, meconium aspiration syndrome, hypoglycemia, neonatal pneumonia, neonatal hyperbilirubinemia, and ABO hemolytic disease. All above perinatal outcomes were registered in the hospital information system by obstetricians in each hospital and confirmed by a senior obstetrician or neonatologist.

### Statistical analysis

Continuous variables are presented as means ± standard deviation (SD), and categorical variables as frequencies and percentages. Estimated fetal weight (EFW) was calculated based on HC, AC, and FDL using the Hadlock formula 3 [23]. Ultrasound measurements were used to model fetal size curves for the ultrasound biometric parameters (AC, HC, BPD, and FDL) and EFW. Log–transformation was applied to biometric parameters and EFW to stabilize variance across gestational ages and improve normal approximations for the error structures. We fitted a linear mixed model with cubic splines for each log–transformed biometric parameter and EFW. Three knots at the 25th, 50th, and 75th percentiles were selected according to the gestational age that ensured an even data distribution [24]. We adjusted the linear mixed model for maternal age, parity, pregravid weight, height, ethnic group (Han vs. minority), education (primary school and below, junior high school, senior high school or equivalent, bachelor’s degree, master’s degree or above), and gender of the infant (male vs. female). We used multiple imputation (with 20 imputations) to impute missing covariate data [25].

Fetal growth velocity contained average velocity and instantaneous velocity. Considering that the interval between two adjacent ultrasound examinations in our data exceeded 4 weeks, it was not suitable for calculating the average velocity. Therefore, we chose to calculate the first derivative of fetal size curves to obtain the instantaneous velocity at each given time point. There are two methods to calculate the first derivatives, the first is to derive the derivatives equation, and the second is to approximately estimate the first derivatives. We described the two methods in Table S3 in detail. We used the second method to obtain the instantaneous velocity. According to the previous literature, both size curves [11] and velocity curves [19] are conditional normal distributions. Therefore, we deduced that the percentile of velocity curves corresponded to the percentile of size curves and then percentiles of fetal growth velocity were obtained based on the exponentiations of the predicted mean and percentiles (the predicted mean and its percentiles were the logarithmic estimates of the original scaled measurements) of the fetal size curves. We divided the entire pregnancy into 2,800 intervals from 12 to 40 gestational weeks, with each interval representing 0.01 gestational week. Velocity was obtained by calculating the increment during each interval [i.e., EFW velocity at each interval (g/week) = EFW increment / 0.01 week].

We used the method introduced by Grantz et al. in the National Institute of Child Health and Human Development (NICHD) fetal growth study [18] to calculate the EFW and AC velocity between the last two ultrasound measurements before delivery. Logistic regression was used to estimate the associations of fetal growth and fetal growth velocity with adverse perinatal outcomes. We adjusted for maternal age, pregravid body mass index (BMI) and parity in multiple comparisons. We also conducted a sensitivity analysis to compare the associations between fetal growth velocity (EFW and AC velocity) in the bottom 10th percentile and adverse perinatal outcomes (neonatal complications, admission to the NICU, PROM, preterm birth < 37 weeks) among the SGA, AGA, and LAG groups. All analyses were performed using SAS software (version 9.4; SAS Institute, Cary, NC, USA). All statistical tests were two–tailed, with *p* values < 0.05 considered significant.

## Results

### Maternal sociodemographic characteristics and perinatal outcomes

A total of 11,891 pregnant women were initially enrolled in the study, of whom 2816 (23.7%) were subsequently excluded based on the exclusion criteria. Thus, 9075 pregnant women with 31,700 ultrasound records (numbers of ultrasound measurements at each gestational week are shown in Figure S1) were included in the final analysis. The characteristics and perinatal outcomes of the pregnant women are shown in Table S2. Their average age was 29.5 ± 4.0 years. The average height was 161.2 ± 4.9 cm and the pregravid weight was 55.5 ± 8.4 kg. Three–quarters of the pregnant women had a bachelor’s degree or above. The average gestational age at delivery was 39.5 ± 1.2 weeks, and the average birth weight was 3318.0 ± 407.6 g. The proportion of pregnant women who delivered before 37 gestational weeks was 2.5% (222/9075). A total of 1695 women (18.7%) experienced rupture of the amniotic sac before delivery, while only 1.1% (97/9075) experienced rupture before 37 weeks.

### Fetal growth velocity

**Fig. 1:**
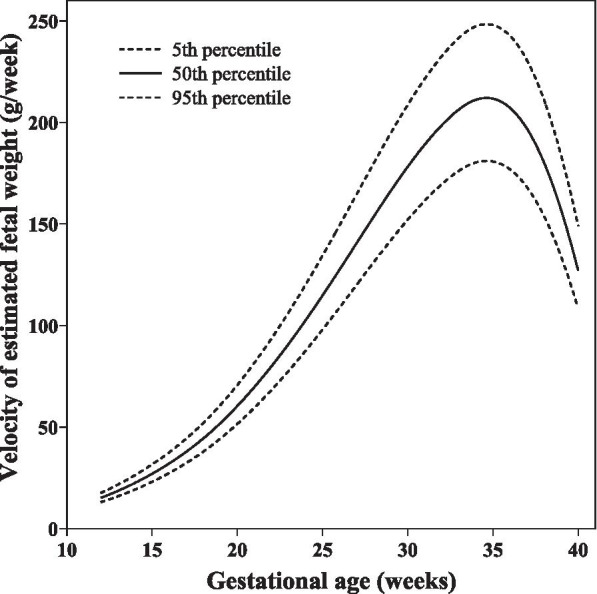
Smooth curve for fetal growth velocity of estimated fetal weight (g/week) according to gestational age

**Fig. 2:**
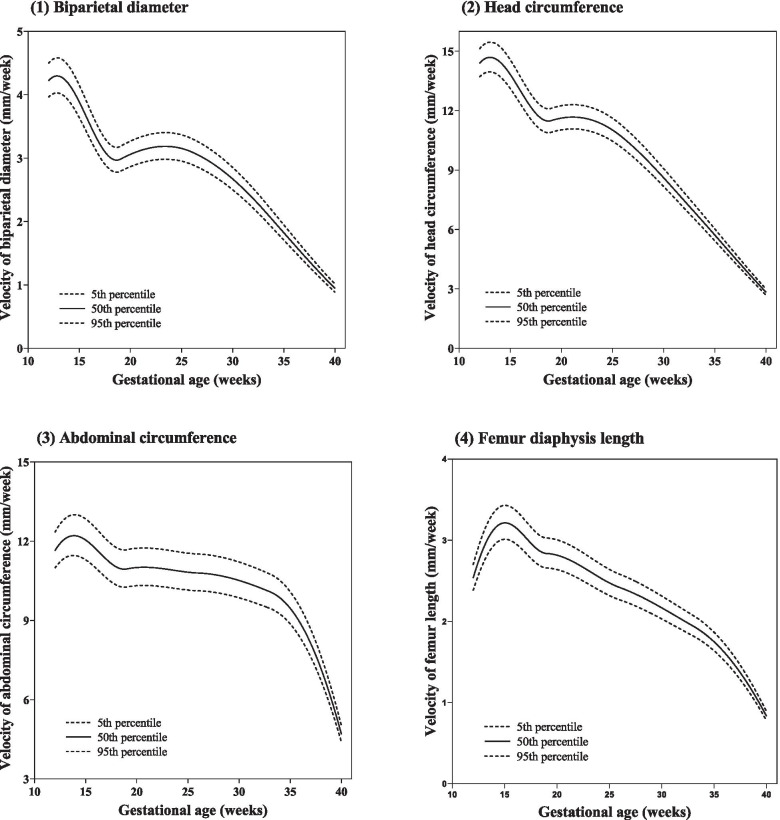
Smooth curve for fetal growth velocity of biparietal diameter, head circumference, abdominal circumference, and femur diaphysis length (mm/week) according to gestational age

**Fig. 3:**
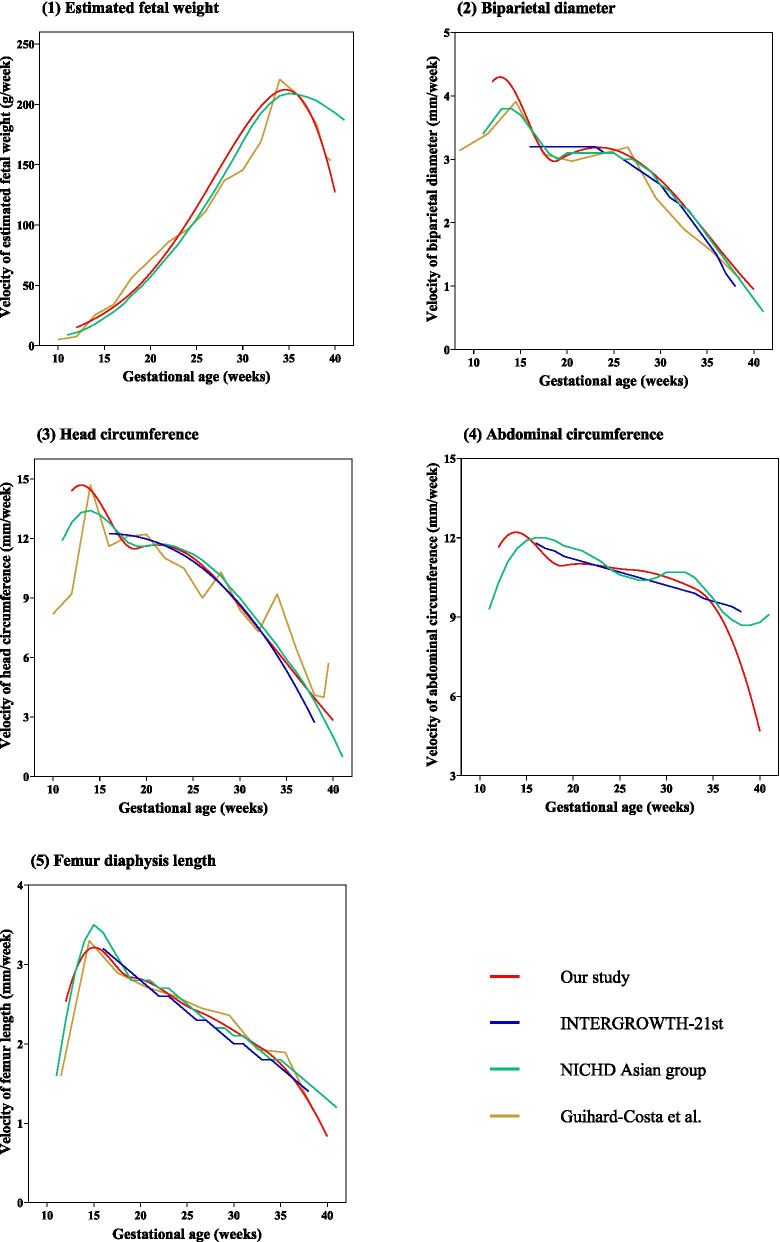
Comparisons of the median velocity between our study and previous studies

The EFW velocity increased from 15.15 g/week at 12 gestational weeks to a peak of 211.71 g/week at 35 gestational weeks, followed by a decrease to 127.04 g/week at the end of pregnancy (Table 1 and Fig. 1). The trajectories of the AC, FDL, HC, and BPD velocities are shown in Tables 2, 3, 4 and 5 and Fig. 2. The BPD and HC velocities both peaked at 13 gestational weeks, decreased from 13 to 19 gestational weeks, and reaccelerated from 19 gestational weeks. The second acceleration of BPD continued until 24 gestational weeks and then steadily decreased to 40 gestational weeks. HC only showed a second acceleration for 2 weeks, followed by a steady decrease from 21 to 40 gestational weeks. AC only showed one period of accelerated growth and, after peaking at 14 gestational weeks, continued to decrease until the end of pregnancy. AC velocity exhibited a small but consistent decrease from 18 to 32 gestational weeks, followed by a sharp decline. FDL also accelerated for 3 weeks; it peaked at 15 gestational weeks and decreased thereafter until 40 gestational weeks. We compared the median fetal velocity curves of our study with those of several previous studies (Fig. 3), including the NICHD fetal growth study [18], INTERGROWTH–21st project [19], and Guihard–Costa et al. [15, 16].

**Table 1:** Percentile for fetal growth velocity of estimated fetal weight (g/week) according to gestational age

Gestational age (weeks)	3rd	5th	10th	25th	50th	75th	90th	95th	97th
12	12.76	13.04	13.48	14.25	15.15	16.11	17.02	17.59	17.98
13	15.58	15.93	16.48	17.45	18.59	19.80	20.97	21.69	22.18
14	18.83	19.25	19.93	21.12	22.52	24.02	25.45	26.35	26.95
15	22.54	23.06	23.87	25.30	27.00	28.80	30.53	31.61	32.33
16	26.77	27.38	28.35	30.05	32.06	34.21	36.26	37.55	38.41
17	31.56	32.29	33.43	35.44	37.81	40.34	42.76	44.28	45.29
18	37.03	37.88	39.22	41.57	44.36	47.32	50.16	51.95	53.14
19	43.29	44.28	45.86	48.61	51.86	55.34	58.66	60.74	62.13
20	50.38	51.53	53.36	56.57	60.35	64.39	68.26	70.68	72.30
21	58.17	59.51	61.62	65.32	69.69	74.35	78.82	81.61	83.48
22	66.66	68.19	70.61	74.85	79.86	85.20	90.31	93.52	95.66
23	75.80	77.54	80.29	85.11	90.81	96.88	102.70	106.35	108.78
24	85.53	87.49	90.60	96.04	102.47	109.33	115.89	120.01	122.76
25	95.78	97.97	101.45	107.55	114.75	122.43	129.78	134.39	137.47
26	106.43	108.87	112.74	119.51	127.51	136.05	144.22	149.34	152.76
27	117.31	120.00	124.26	131.72	140.54	149.94	158.95	164.59	168.36
28	128.16	131.09	135.75	143.90	153.53	163.80	173.63	179.80	183.92
29	138.72	141.90	146.94	155.76	166.18	177.30	187.94	194.62	199.08
30	148.72	152.13	157.53	166.99	178.16	190.09	201.50	208.66	213.44
31	157.86	161.48	167.21	177.25	189.11	201.77	213.89	221.48	226.56
32	165.81	169.61	175.63	186.18	198.64	211.93	224.66	232.64	237.97
33	172.24	176.19	182.45	193.40	206.34	220.15	233.37	241.66	247.20
34	176.26	180.30	186.71	197.91	211.16	225.29	238.82	247.30	252.96
35	176.72	180.77	187.19	198.43	211.71	225.88	239.45	247.95	253.63
36	172.94	176.91	183.19	194.19	207.19	221.05	234.33	242.65	248.21
37	164.33	168.09	174.06	184.51	196.86	210.03	222.64	230.55	235.83
38	150.42	153.87	159.33	168.89	180.19	192.25	203.79	211.03	215.86
39	130.95	133.96	138.72	147.05	156.91	167.42	177.48	183.79	188.01
40	105.88	108.33	112.21	119.00	127.04	135.61	143.83	148.98	152.42

**Table 2:** Percentile for fetal growth velocity of biparietal diameter (mm/week) according to gestational age

Gestational age (weeks)	3rd	5th	10th	25th	50th	75th	90th	95th	97th
12	3.93	3.96	4.02	4.11	4.22	4.33	4.43	4.50	4.54
13	3.99	4.02	4.08	4.18	4.29	4.41	4.51	4.58	4.62
14	3.86	3.90	3.96	4.05	4.16	4.28	4.38	4.44	4.49
15	3.60	3.64	3.69	3.78	3.89	3.99	4.09	4.15	4.19
16	3.29	3.32	3.37	3.45	3.54	3.64	3.73	3.79	3.82
17	2.99	3.02	3.06	3.14	3.23	3.31	3.39	3.44	3.48
18	2.79	2.82	2.86	2.93	3.01	3.10	3.17	3.22	3.25
19	2.77	2.79	2.83	2.90	2.98	3.06	3.14	3.19	3.22
20	2.84	2.87	2.91	2.98	3.06	3.14	3.22	3.27	3.30
21	2.90	2.92	2.97	3.04	3.12	3.21	3.29	3.33	3.36
22	2.93	2.96	3.01	3.08	3.16	3.25	3.33	3.38	3.41
23	2.95	2.98	3.03	3.10	3.18	3.27	3.35	3.40	3.43
24	2.95	2.98	3.02	3.10	3.18	3.27	3.35	3.40	3.43
25	2.92	2.95	3.00	3.07	3.15	3.24	3.32	3.37	3.40
26	2.87	2.90	2.94	3.01	3.10	3.18	3.26	3.31	3.34
27	2.80	2.82	2.87	2.94	3.02	3.10	3.18	3.22	3.25
28	2.71	2.73	2.77	2.84	2.92	3.00	3.07	3.12	3.15
29	2.60	2.62	2.66	2.73	2.80	2.88	2.95	2.99	3.02
30	2.48	2.50	2.54	2.60	2.67	2.74	2.81	2.85	2.88
31	2.34	2.36	2.40	2.46	2.52	2.59	2.66	2.69	2.72
32	2.19	2.21	2.24	2.30	2.36	2.43	2.49	2.52	2.55
33	2.03	2.05	2.08	2.13	2.19	2.25	2.30	2.34	2.36
34	1.86	1.88	1.91	1.95	2.01	2.06	2.11	2.14	2.16
35	1.69	1.71	1.73	1.77	1.82	1.87	1.92	1.95	1.96
36	1.52	1.53	1.56	1.60	1.64	1.68	1.72	1.75	1.77
37	1.35	1.36	1.38	1.42	1.46	1.50	1.53	1.56	1.57
38	1.19	1.20	1.22	1.25	1.28	1.32	1.35	1.37	1.38
39	1.03	1.04	1.05	1.08	1.11	1.14	1.17	1.19	1.20
40	0.88	0.88	0.90	0.92	0.95	0.97	1.00	1.01	1.02

**Table 3:** Percentile for fetal growth velocity of head circumference (mm/week) according to gestational age

Gestational age (weeks)	3rd	5th	10th	25th	50th	75th	90th	95th	97th
12	13.60	13.70	13.85	14.10	14.39	14.68	14.95	15.12	15.22
13	13.85	13.96	14.11	14.38	14.69	15.00	15.28	15.45	15.57
14	13.62	13.72	13.88	14.14	14.45	14.76	15.05	15.22	15.33
15	13.02	13.12	13.27	13.53	13.82	14.12	14.40	14.56	14.67
16	12.24	12.33	12.47	12.72	13.00	13.28	13.54	13.69	13.80
17	11.48	11.57	11.70	11.93	12.19	12.45	12.70	12.84	12.94
18	10.94	11.02	11.15	11.37	11.62	11.87	12.10	12.24	12.34
19	10.82	10.91	11.03	11.25	11.49	11.74	11.97	12.11	12.20
20	10.94	11.03	11.16	11.37	11.62	11.87	12.11	12.25	12.34
21	10.99	11.07	11.20	11.42	11.67	11.92	12.16	12.30	12.39
22	10.96	11.04	11.17	11.39	11.64	11.89	12.12	12.26	12.36
23	10.85	10.93	11.06	11.27	11.52	11.77	12.00	12.14	12.23
24	10.65	10.73	10.86	11.07	11.31	11.56	11.78	11.92	12.01
25	10.37	10.45	10.57	10.78	11.02	11.26	11.48	11.61	11.70
26	10.01	10.09	10.21	10.41	10.63	10.86	11.08	11.21	11.29
27	9.59	9.66	9.77	9.97	10.18	10.40	10.61	10.73	10.81
28	9.12	9.19	9.30	9.48	9.69	9.90	10.09	10.21	10.29
29	8.62	8.69	8.79	8.96	9.16	9.36	9.54	9.65	9.72
30	8.10	8.16	8.26	8.42	8.60	8.79	8.96	9.07	9.14
31	7.56	7.62	7.71	7.86	8.03	8.21	8.37	8.47	8.53
32	7.02	7.07	7.16	7.30	7.45	7.62	7.77	7.86	7.92
33	6.48	6.53	6.60	6.73	6.88	7.03	7.17	7.25	7.30
34	5.93	5.98	6.05	6.17	6.30	6.44	6.56	6.64	6.69
35	5.38	5.42	5.48	5.59	5.71	5.84	5.95	6.02	6.07
36	4.82	4.86	4.92	5.01	5.12	5.23	5.34	5.40	5.44
37	4.27	4.30	4.35	4.44	4.53	4.63	4.72	4.78	4.82
38	3.72	3.75	3.80	3.87	3.95	4.04	4.12	4.17	4.20
39	3.19	3.21	3.25	3.31	3.38	3.46	3.53	3.57	3.59
40	2.66	2.68	2.71	2.77	2.83	2.90	2.95	2.99	3.01

**Table 4:** Percentile for fetal growth velocity of abdominal circumference (mm/week) according to gestational age

Gestational age (weeks)	3rd	5th	10th	25th	50th	75th	90th	95th	97th
12	10.89	10.98	11.13	11.37	11.64	11.92	12.18	12.34	12.44
13	11.27	11.37	11.53	11.79	12.09	12.40	12.69	12.86	12.97
14	11.35	11.46	11.62	11.89	12.21	12.53	12.82	13.00	13.12
15	11.19	11.29	11.46	11.73	12.04	12.36	12.66	12.84	12.96
16	10.88	10.98	11.14	11.40	11.71	12.02	12.31	12.49	12.60
17	10.52	10.62	10.77	11.03	11.32	11.63	11.91	12.08	12.19
18	10.24	10.34	10.49	10.74	11.02	11.32	11.59	11.76	11.87
19	10.17	10.26	10.41	10.66	10.95	11.24	11.51	11.68	11.78
20	10.22	10.32	10.46	10.72	11.00	11.30	11.57	11.73	11.84
21	10.23	10.32	10.47	10.72	11.01	11.30	11.58	11.74	11.85
22	10.20	10.30	10.45	10.70	10.98	11.28	11.55	11.71	11.82
23	10.16	10.25	10.40	10.65	10.93	11.23	11.50	11.66	11.77
24	10.10	10.20	10.34	10.59	10.88	11.17	11.44	11.60	11.71
25	10.06	10.15	10.29	10.54	10.83	11.12	11.38	11.55	11.65
26	10.03	10.12	10.27	10.51	10.79	11.08	11.35	11.51	11.62
27	10.00	10.09	10.24	10.48	10.76	11.05	11.32	11.48	11.59
28	9.95	10.04	10.18	10.43	10.70	10.99	11.26	11.42	11.52
29	9.87	9.96	10.10	10.34	10.62	10.90	11.17	11.33	11.43
30	9.77	9.86	10.00	10.24	10.51	10.79	11.05	11.21	11.31
31	9.65	9.74	9.88	10.11	10.38	10.66	10.92	11.08	11.18
32	9.51	9.60	9.74	9.97	10.24	10.52	10.77	10.92	11.02
33	9.37	9.46	9.59	9.82	10.09	10.36	10.61	10.76	10.86
34	9.15	9.24	9.37	9.59	9.85	10.11	10.36	10.51	10.60
35	8.78	8.86	8.99	9.21	9.45	9.71	9.94	10.08	10.18
36	8.25	8.33	8.45	8.65	8.88	9.12	9.34	9.47	9.56
37	7.55	7.62	7.73	7.91	8.12	8.34	8.54	8.66	8.75
38	6.67	6.73	6.82	6.99	7.17	7.37	7.54	7.65	7.72
39	5.60	5.65	5.73	5.87	6.03	6.19	6.34	6.43	6.49
40	4.34	4.39	4.45	4.56	4.69	4.82	4.94	5.01	5.06

**Table 5:** Percentile for fetal growth velocity of femur diaphysis length (mm/week) according to gestational age

Gestational age (weeks)	3rd	5th	10th	25th	50th	75th	90th	95th	97th
12	2.36	2.38	2.41	2.47	2.54	2.60	2.66	2.70	2.72
13	2.70	2.73	2.77	2.83	2.91	2.99	3.06	3.10	3.13
14	2.91	2.94	2.98	3.06	3.14	3.22	3.30	3.35	3.38
15	2.98	3.01	3.06	3.13	3.21	3.30	3.38	3.43	3.46
16	2.94	2.96	3.01	3.08	3.16	3.25	3.33	3.37	3.41
17	2.82	2.84	2.89	2.96	3.04	3.12	3.19	3.24	3.27
18	2.69	2.72	2.76	2.83	2.90	2.98	3.05	3.10	3.13
19	2.63	2.66	2.70	2.76	2.84	2.91	2.98	3.03	3.06
20	2.61	2.64	2.68	2.74	2.82	2.89	2.96	3.01	3.03
21	2.57	2.60	2.63	2.70	2.77	2.84	2.91	2.96	2.98
22	2.51	2.53	2.57	2.63	2.70	2.78	2.84	2.88	2.91
23	2.44	2.46	2.50	2.56	2.63	2.70	2.76	2.80	2.83
24	2.37	2.39	2.42	2.48	2.55	2.62	2.68	2.72	2.74
25	2.30	2.32	2.35	2.41	2.47	2.54	2.60	2.64	2.67
26	2.24	2.27	2.30	2.35	2.42	2.48	2.54	2.58	2.60
27	2.20	2.22	2.25	2.30	2.37	2.43	2.49	2.52	2.55
28	2.14	2.16	2.19	2.25	2.31	2.37	2.43	2.46	2.48
29	2.08	2.10	2.13	2.18	2.24	2.30	2.36	2.39	2.41
30	2.01	2.03	2.06	2.11	2.17	2.23	2.28	2.31	2.33
31	1.94	1.96	1.99	2.04	2.09	2.15	2.20	2.23	2.25
32	1.87	1.89	1.92	1.96	2.02	2.07	2.12	2.15	2.17
33	1.80	1.82	1.85	1.89	1.94	2.00	2.04	2.07	2.09
34	1.72	1.74	1.77	1.81	1.86	1.91	1.95	1.98	2.00
35	1.62	1.64	1.66	1.70	1.75	1.80	1.84	1.87	1.88
36	1.50	1.51	1.54	1.57	1.62	1.66	1.70	1.72	1.74
37	1.35	1.37	1.38	1.42	1.46	1.50	1.53	1.55	1.57
38	1.18	1.19	1.21	1.24	1.27	1.31	1.34	1.36	1.37
39	0.99	1.00	1.01	1.04	1.06	1.09	1.12	1.14	1.15
40	0.77	0.78	0.79	0.81	0.83	0.86	0.88	0.89	0.90

### Associations of fetal growth and fetal growth velocity with adverse perinatal outcomes

**Fig. 4:**
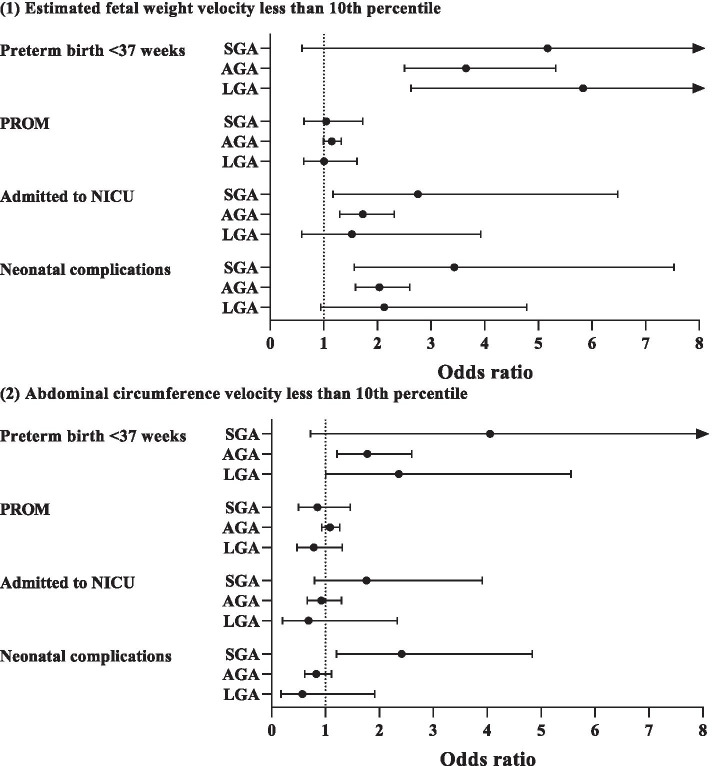
Sensitivity analyses of the association between fetal growth velocity and adverse perinatal outcomes. PROM, premature rupture of membranes; NICU, neonatal intensive care unit; SGA, small–for–gestational age; AGA, appropriate–for–gestational age; LGA, large–for–gestational age

Table 6 shows the associations of fetal growth and fetal growth velocity with adverse perinatal outcomes. Traditional fetal size curves and fetal growth velocity curves both had certain advantages for indicating the risk of adverse perinatal outcomes. EFW and AC in the bottom 10th percentile, as defined by traditional fetal size curves, had stronger relationships with both SGA and admittance to the NICU. EFW velocity in the bottom 10th percentile had higher risks of neonatal complications (odds ratio [OR] = 2.23, 95% confidence interval [CI]: 1.79–2.78) and preterm birth < 37 weeks (OR = 3.68, 95% CI: 2.64–5.14). AC velocities in the bottom 10th percentile were associated with SGA (OR = 1.57, 95% CI: 1.27–1.92) and preterm birth < 37 weeks (OR = 1.84, 95% CI: 1.31–2.58). Sensitivity analyses (Fig. 4 and Figure S2) showed that EFW velocity in the bottom 10th percentile higher risk of adverse pregnancy outcomes in the AGA group, which were significantly associated with neonatal complications (OR = 2.03, 95% CI: 1.59–2.60), admission to the NICU (OR = 1.73, 95% CI: 1.29–2.31), and preterm birth < 37 weeks (OR = 3.65, 95% CI: 2.50–5.32). In contrast, using only one biometric parameter could not present the advantages of the AGA group.

**Table 6:** Association between adverse perinatal outcomes of fetal growth size less than 10th percentile and fetal growth velocity less than 10th percentile

Outcomes	SGA		Neonatal complications		Admitted to NICU		PROM		Preterm birth < 37 weeks	
	OR (95%CI)	*P* value	OR (95%CI)	*P* value	OR (95%CI)	*P* value	OR (95%CI)	*P* value	OR (95%CI)	*P* value
EFW < 10th	10.44 (7.93–13.76)	< 0.01	1.79 (1.15–2.80)	0.01	2.28 (1.42–3.67)	< 0.01	0.90 (0.65–1.25)	0.55	1.85 (0.96–3.56)	0.07
AC < 10th	11.87 (8.59–16.41)	< 0.01	1.80 (1.05–3.10)	0.03	2.06 (1.12–3.76)	0.02	0.80 (0.53–1.21)	0.29	1.60 (0.70–3.68)	0.27
BPD < 10th	3.62 (2.48–5.30)	< 0.01	2.61 (1.66–4.11)	< 0.01	3.26 (2.01–5.29)	< 0.01	0.94 (0.64–1.38)	0.75	1.95 (0.94–4.04)	0.07
HC < 10th	3.24 (2.24–4.70)	< 0.01	1.77 (1.08–2.91)	0.02	2.57 (1.56–4.25)	< 0.01	0.82 (0.56–1.19)	0.30	1.79 (0.87–3.70)	0.12
FL < 10th	3.57 (2.09–6.10)	< 0.01	1.04 (0.42–2.59)	0.93	1.48 (0.60–3.69)	0.40	1.02 (0.60–1.74)	0.93	2.13 (0.77–5.89)	0.15
EFW velocity < 10th	2.19 (1.80–2.66)	< 0.01	2.23 (1.79–2.78)	< 0.01	1.86 (1.44–2.41)	< 0.01	1.11 (0.98–1.27)	0.11	3.68 (2.64–5.14)	< 0.01
AC velocity < 10th	1.57 (1.27–1.92)	< 0.01	0.97 (0.75–1.26)	0.83	1.01 (0.75–1.36)	0.93	1.03 (0.89–1.19)	0.69	1.84 (1.31–2.58)	< 0.01
BPD velocity < 10th	1.32 (1.05–1.67)	0.02	0.74 (0.54–1.02)	0.06	0.84 (0.59–1.20)	0.34	1.04 (0.88–1.22)	0.65	1.27 (0.85–1.89)	0.24
HC velocity < 10th	1.22 (0.97–1.54)	0.09	0.83 (0.62–1.11)	0.22	1.04 (0.76–1.44)	0.80	1.10 (0.94–1.29)	0.22	0.99 (0.65–1.50)	0.97
FL velocity < 10th	1.22 (0.97–1.52)	0.09	0.84 (0.63–1.12)	0.24	1.25 (0.92–1.68)	0.15	1.13 (0.97–1.31)	0.11	1.10 (0.75–1.63)	0.62

*EFW* estimated fetal weight, *AC* abdominal circumference, *PROM* premature rupture of membranes, *NICU* neonatal intensive care unit, *SGA* small–for–gestational age, *OR* odds ratio, *CI* confidence interval

## Discussion

To our knowledge, this study is the first to attempt to develop a fetal growth velocity chart specific to the Chinese population. We established fetal growth velocity reference charts for EFW, AC, FDL, HC, and BPD according to gestational weeks. EFW velocity was shown to increase beginning at 12 gestational weeks, reaching a maximum velocity of 211.71 g/week at 35 gestational weeks, followed by a gradual decrease to 127.04 g/week at 40 gestational weeks. The other four biometric parameters showed similar patterns characterized by accelerations in the early second trimester peaking at 13 gestational weeks for BPD and HC, 14 gestational weeks for AC, and 15 gestational weeks for FDL. BPD and HC experienced a second acceleration in the mid and late second trimester, at 19–24 and 19–21 gestational weeks, respectively. Compared to traditional fetal size curves, we found that fetal growth velocities in the bottom 10th percentile provided more information associated with neonatal complications and preterm birth < 37 weeks.

EFW velocity was previously reported by the NICHD fetal growth study [18] in a US population, and by Guihard–Costa et al. [16] in a French population; the median EFW velocity patterns were similar to our findings. Both studies observed that EFW velocity peaked at around 35 gestational weeks, with maximum velocities of 220.66 and 209 g/week, respectively. However, after 36 gestational weeks, the EFW velocity of the NICHD fetal growth study decreased slower than in our study, resulting in a higher EFW velocity at the end of pregnancy relative to this study. This difference in third trimester EFW velocity may be due to the fact that the pregravid BMI of our Chinese mothers was lower than that of Asian American mothers, which limited fetal growth in the third trimester [26, 27].

In general, the patterns of BPD, HC, AC, and FDL velocities in our findings were consistent with previous studies. The NICHD fetal growth study and Guihard–Costa et al. reported acceleration of BPD, HC, and FDL velocities in the early second trimester, with peaks around 13–15 gestational weeks. However, the BPD velocity in the early second trimester reported here was higher than in both of the previous studies. The maximum velocity of HC was similar to Guihard–Costa et al., and higher than that of the NICHD fetal growth study, while the NICHD fetal growth study had the highest maximum velocity of FDL. The INTERGROWTH–21st project reported that fetal growth velocities from 16 gestational weeks, so the first acceleration seen in our study was not present in theirs; otherwise, the fetal growth velocities were similar, except for AC velocity. In our study, AC velocity decreased rapidly during the late third trimester, from 10.09 mm/week at 33 weeks to 4.69 mm/week at 40 weeks. In contrast, the AC velocity in the NICHD fetal growth study and INTERGROWTH–21st project did not exhibit this sharp decrease in the third trimester, and in fact showed a third acceleration after 38 weeks in the case of the NICHD fetal growth study. In addition to these above publications, two other studies also reported fetal growth velocities. Bertino et al. [14] reported the velocities of AC, FDL, HC, and BPD in an Italian population in 1996, which showed similar velocity curves to our population. However, their curves only showed one period of acceleration and peaked later than those of our participants. Fescina et al. [28] reported that, in a Latin American population, BPD velocity peaked at 13 weeks, decreased at around 15 weeks, stabilized from 15 to 30 weeks, and decreased again after 30 weeks [29]. We speculate that differences in fetal growth velocity can be partially explained by differences in genetic background and living environment between study populations. Higher AC and EFW velocities may be related to higher pregravid BMI in some populations [30, 31]; however, we note that epidemiological surveys showed that, due to lower maternal pregravid BMI, Chinese babies had lower birth weights than American and Chinese American babies [26, 27]. Furthermore, these studies employed different ultrasound equipment, participant inclusion criteria, and modeling methods, all of which may have affected fetal growth velocity trajectories.

Our findings showed that, compared to traditional fetal size curves, fetal growth velocity curves could provide additional information for associated with neonatal complications and preterm birth < 37 weeks. Moreover, sensitivity analysis showed that EFW velocity in the bottom 10th percentile was significantly associated with more adverse perinatal outcomes (including preterm birth < 37 weeks, admitted to NICU, neonatal complications) in the AGA group than the LGA and SGA groups. Hendrix et al. [32] reported similar findings, i.e., decreased fetal growth velocities between around 20 and 32 weeks were significantly associated with adverse neonatal outcomes (neonatal asphyxia, sepsis, respiratory distress syndrome, and transient shortness of breath) in AGA neonates. In a prospective cohort of 3977 pregnant women, Sovio et al. [33] found that, compared to EFW velocity alone in the bottom 10th percentile, AC velocity in the lowest decile was also associated with a higher relative risk of SGA and other adverse perinatal outcomes. Deter et al. [34], in a retrospective observational study of 126 pregnant women, found that the AC velocity of fetuses with restricted third–trimester growth was significantly lower than that of fetuses with normal growth. These reduced fetal growth velocities may indicate placental insufficiency, with both Kennedy et al. [35] and MacDonald et al. [36] reporting that reduced EFW and AC velocity in the third trimester was associated with a cerebroplacental ratio < 5th percentile at 36 gestational weeks, neonatal acidosis (umbilical artery pH < 7.15 at birth), and low neonatal body fat percentage (< 4.2% for males and < 5.8% for females). Summarizing our findings and the above–mentioned studies, fetal growth velocity curves can provide additional information for obstetric clinical practice, which could aid the identification of potentially high–risk AGA neonates.

## Limitations

There were some limitations to the present study. First, as it relied on real–world clinical data, the ultrasound examinations were non–uniformly distributed from 12 to 40 gestational weeks, with most pregnant women receiving three ultrasound examinations at around 22, 30, and 37 gestational weeks (consistent with the national policy of antenatal care in China [37], which recommends at least three ultrasound examinations for fetal anthropometry at the above times points). Therefore, the interval between two adjacent ultrasound examinations for most pregnant women exceeded 4 weeks, such that the fetal growth velocities calculated for the last two ultrasound examinations may have been slower than the actual velocities. Considering the above issues, we chose to calculate the derivative of the percentiles of the growth curve to obtain the percentiles of the velocity curves. The advantage of this method is that it can make full use of the data in our cohort and can estimate the instantaneous velocity at a given time point. However, in clinical practice, the instantaneous velocity is difficult to obtain, obstetricians usually calculate the average speed in 2 weeks for pregnancy monitoring, which is somewhat different from the velocity references in our study.

## Conclusions

Here, we presented the first fetal growth velocity charts specific to the Chinese population. Our findings revealed modest differences in fetal growth velocities between Chinese populations and other populations. We recommend the utilization of fetal growth standards, including growth velocity curves, designed specifically for the population to which they are applied. More importantly, we found that fetal growth velocity lower than the 10th percentile provided more information to understand the risk of adverse perinatal outcomes, especially in AGA neonates. Finally, we suggest that future researchers consider adding fetal growth velocity to existing multivariable models to improve the accuracy of predictions of adverse perinatal outcomes.

## Supplementary Information

**Additional file 1: Table S1.** List of partner hospitals in this study. **Table S2.** Demographic characteristics of participants. **Figure S1.** Numbers of ultrasound measurements at each gestational week. **Figure S2.** Sensitivity analyses of the association between fetal growth velocity and adverse perinatal outcomes. **Table S3.** Equation of linear mixed model with cubic splines and calculation of first derivatives.

## Data Availability

The datasets generated and/or analyzed during the current study are not publicly available due to local ownership of the data, but are accessible from the corresponding authors (Prof. X Wang, xlwang@bjmu.edu.cn; and Prof. Y Wei, weiyuanbysy@163.com) on reasonable request.

## Abbreviations

EFW: Estimated fetal weight
BPD: Biparietal diameter
AC: Abdominal circumference
HC: Head circumference
FL: Femur length
SGA: Small–for–gestational age
FGR: Fetal growth restriction
NICU: Neonatal intensive care unit
PROM: Premature rupture of membranes
LGA: Large–for–gestational age
AGA: Appropriate–for–gestational age
SD: Standard deviation
BMI: Body mass index
