# Supplementary material for: Fetal growth velocity references from a Chinese population–based fetal growth study
Source: BMC Pregnancy Childbirth. 2021 Oct 9;21:688. doi: 10.1186/s12884-021-04149-x (PMC8501691; doi:10.1186/s12884-021-04149-x)
Supplement: Supplementary file 1 — Additional file 1: Table S1. List of partner hospitals in this study. Table S2. Demographic characteristics of participants. Figure S1. Numbers of ultrasound measurements at each gestational week. Figure S2. Sensitivity analyses of the association between fetal growth velocity and adverse perinatal outcomes. Table S3. Equation of linear mixed model with cubic splines and calculation of first derivatives. [file 12884_2021_4149_MOESM1_ESM.pdf]

Fetal growth velocity references from a Chinese population–based fetal growth study

Supplementary materials

Content

Table S1 List of partner hospitals in this study 1
Table S2 Demographic characteristics of participants 2
Figure S1 Numbers of ultrasound measurements at each gestational week 3
Figure S2 Sensitivity analyses of the association between fetal growth velocity and adverse perinatal outcomes..... 4
Table S3 Equation of linear mixed model with cubic splines and calculation of first derivatives 5

Table S1 List of partner hospitals in this study

Provinces	Hospitals
Beijing	Tongzhou Maternal and Child Health Hospital
Beijing	Peking University Third Hospital
Liaoning	Shenyang Maternity and Infant Hospital
Liaoning	Shengjing Hospital Affiliated to China Medical University
Hebei	Shijiazhuang Maternal and Child Health Hospital
Hebei	Qinhuangdao Maternal and Child Health Hospital
Shanxi	Children’s Hospital of Shanxi, Women Health Center of Shanxi
Shanxi	Changzhi Maternal and Child Health Hospital
Jilin	Changchun Maternity Hospital
Gansu	Gansu Maternal and Child Health Hospital
Inner Mongolia Autonomous Region	Inner Mongolia Maternal and Child Health Hospital
Shandong	Dongchangfu Maternal and Child Health Hospital
Henan	Zhengzhou Central Hospital
Xinjiang Uygur Autonomous Region	Urumqi Maternal and Child Health Hospital
Shaanxi	Northwest Women's and Children's Hospital
Guangdong	The Third Affiliated Hospital of Guangzhou Medical University
Guangxi Zhuang Autonomous Region	Maternal and Child Health Hospital of Guangxi
Hunan	Hunan Maternal and Child Health Hospital
Jiangxi	Jiangxi Maternal and Child Health Hospital
Fujian	Fujian Maternal and Child Health Hospital
Fujian	The First Affiliated Hospital of Xiamen University
Chongqing	Chongqing Maternal and Child Health Hospital
Chongqing	The First Affiliated Hospital of Chongqing Medical University
Hubei	Hubei Maternal and Child Health Hospital

Table S2 Demographic characteristics of participants

Maternal characteristics / Perinatal outcomes	Participants (n=9,075)
Maternal age (years), mean ± SD	29.5±4.0
Height (cm) , mean ± SD	161.2±4.9
Pregravid weight (kg) , mean ± SD	55.5±8.4
Ethnic group, n(%)	
Han	8,451(93.1)
Minority	624(6.9)
Education, n(%)	
Primary school	39(0.4)
Junior high school	777(8.6)
Senior high school	1,455(16.0)
Bachelor degree	6,098(67.2)
Master or above	706(7.8)
Parity, n(%)	
0	5,663(62.4)
≥1	3,412(37.6)
Neonatal gender, n(%)	
Male	4,775(52.6)
Female	4,300(47.4)
Mode of delivery, n(%)	
Spontaneous	5,694(62.7)
Vacuum	62(0.7)
Forceps	124(1.4)
Cesarean section	3,195(35.2)
Premature rupture of membranes, n(%)	1,695(18.7)
Premature, n(%)	222(2.5)
Preterm premature rupture of membranes, n(%)	97(1.1)
Admitted to NICU	335 (3.7)
Neonatal complications	453 (4.99)
Gestational age at delivery, mean ± SD	39.5±1.2
Birth weight , mean ± SD	3,318.0±407.6
Birth length, mean ± SD	50.1±1.8

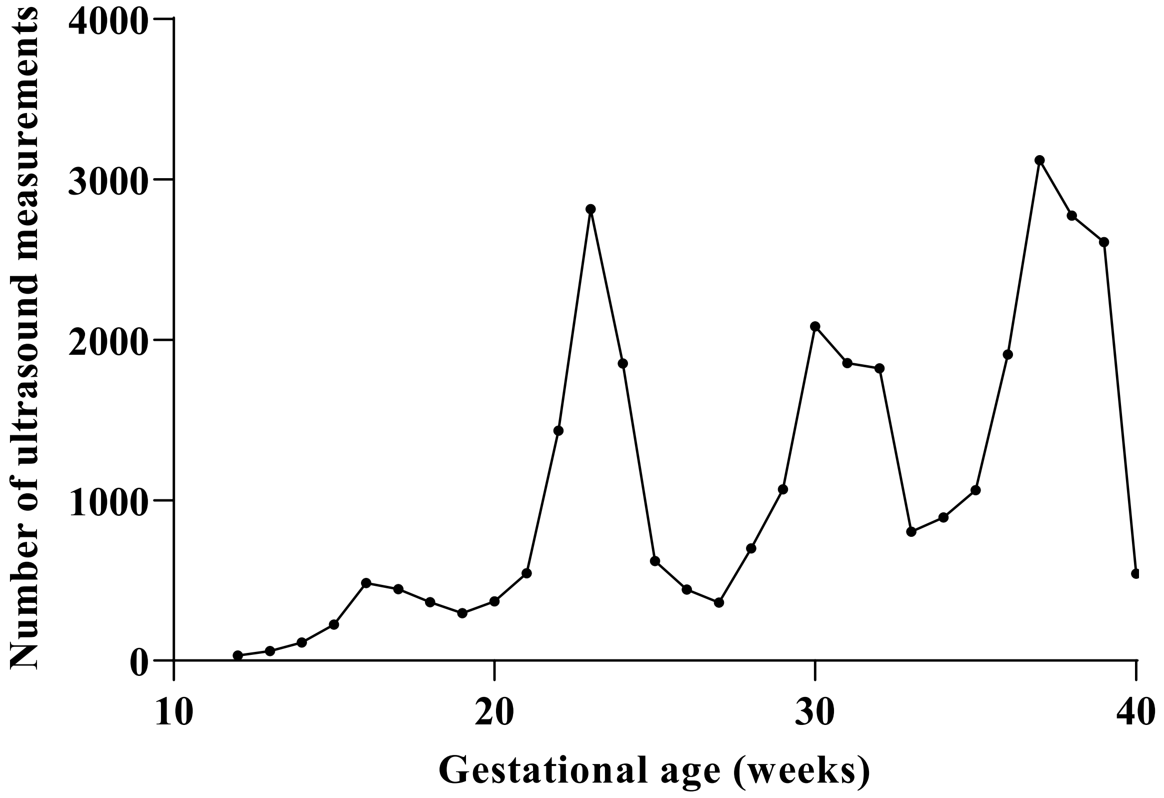

Figure S1 Numbers of ultrasound measurements at each gestational week

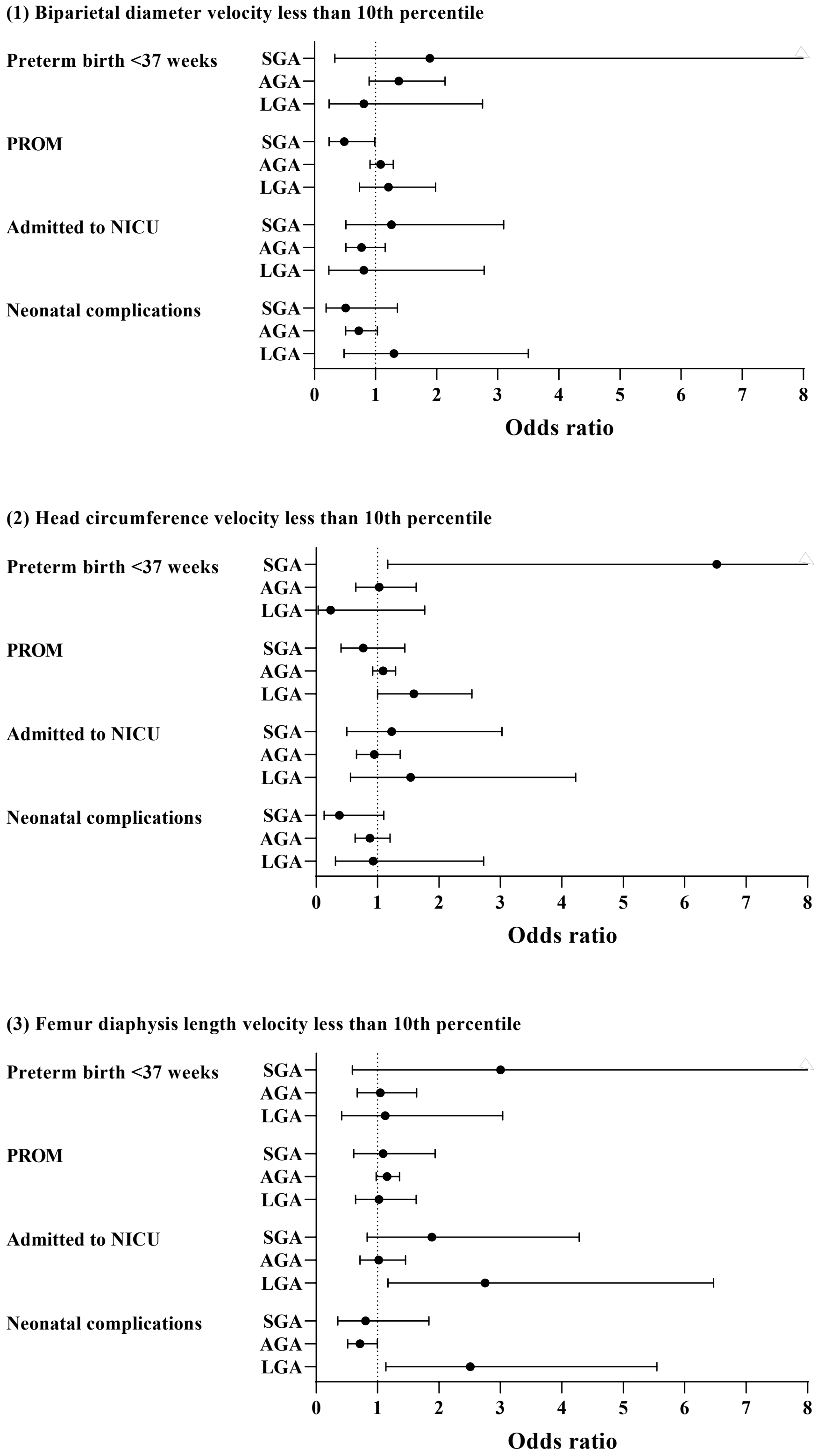

Figure S2 Sensitivity analyses of the association between fetal growth velocity and adverse perinatal outcomes

PROM, premature rupture of membranes; NICU, neonatal intensive care unit; SGA, small–for–gestational age; AGA, appropriate–for–gestational age; LGA, large–for–gestational age.

Table S3 Equation of linear mixed model with cubic splines and calculation of first derivatives

Regression equation	$Y_{ij}^* = \ln(Y_{ij}) = \beta_0 + \beta_1 ges_{ij} + \beta_2 ges_{ij}^2 + \beta_3 ges_{ij}^3 + \beta_4 (ges_{ij} - \xi_1)_+^3 + \beta_5 (ges_{ij} - \xi_2)_+^3 + \beta_6 (ges_{ij} - \xi_3)_+^3 + b_{i0} + b_{i1} ges_{ij} + b_{i2} ges_{ij}^2 + b_{i3} ges_{ij}^3 + \varepsilon_{ij}$ where $(ges_{ij} - \xi_k)_+^3 = \begin{cases} 0 & ges \leq \xi \\ (ges_{ij} - \xi_k)^3 & ges > \xi \end{cases}$ $\varepsilon_{ij} \sim N(0, \sigma^2)$
First derivatives equation	$Y_{ij}' = exp(Y_{ij}^*) \left[\beta_1 + 2\beta_2 ges_{ij} + 3\beta_3 ges_{ij}^2 + 3\beta_4 (ges_{ij} - \xi_1)_+^2 + 3\beta_5 (ges_{ij} - \xi_2)_+^2 + 3\beta_6 (ges_{ij} - \xi_3)_+^2\right]$
Approximate estimation of the first derivative	$\text{Velovity} = \frac{\Delta Y(ges_{ij})}{\Delta t} = \lim_{h \to 0} \frac{Y(ges_{ij+h}) - Y(ges_{ij})}{t_{ij+h} - t_{ij}}$

Regression equation: Y_{ij}^* donates log–transformed ultrasound biometric parameters or EFW; Y_{ij} donates original scale ultrasound biometric parameters or EFW; ges_{ij} donates the *j*th gestational weeks for *i*th pregnant women; $(ges_{ij} - \xi_k)_+^3$ donates the three knot sequence at the 25th, 50th, and 75th percentiles of gestational weeks; $\beta_0 + \beta_1 ges_{ij} + \beta_2 ges_{ij}^2 + \beta_3 ges_{ij}^3 + \beta_4 (ges_{ij} - \xi_1)_+^3 + \beta_5 (ges_{ij} - \xi_2)_+^3 + \beta_6 (ges_{ij} - \xi_3)_+^3$ donates a truncated polynomial splines; $b_{i0} + b_{i1} ges_{ij} + b_{i2} ges_{ij}^2 + b_{i3} ges_{ij}^3$ donates random intercept and random slope. When calculating the average velocity in population, we assume that the average departure of the individual fetal growth velocity relative to the fetal growth velocity in population is zero. Therefore, we only need the derivative of the fixed effects in the regression equation to obtain the velocity equation.

First derivatives equation: Y_{ij}' donates the first derivative of original scale of the velocity of ultrasound biometric parameters or EFW.

Approximate estimation of the first derivative: According to the rigorous definition of the derivative, a value of *h* close to zero gives a good approximation to the slope of the tangent line, and smaller values (in absolute value) of *h* will, in general, give better approximations. We chose 0.01 as the value of *h* in the present study to obtain an approximate estimate of fetal growth velocity. This formula actually calculated the average velocity in an interval of 0.01 weeks, considering that the interval is very small, so we use the average velocity as an approximate estimate of the instantaneous velocity. We found pervious literature reported that both size curves (Am J Obstet Gynecol, 2015,213 (4):449.e441-449.e441.) and velocity curves (Am J Obstet Gynecol, 2021,224 (2):208.e201-208.e218.)

were conditional normal distributions. Therefore, we deduced that the percentile of velocity curves corresponded to the percentile of size curves and then calculated the approximate derivatives (fetal growth increment in 0.01 weeks) of the 3rd, 5th, 10th, 25th, 50th, 75th, 90th, 95th, and 97th percentile of the fetal size curves to obtain the corresponding percentile of fetal growth velocity curves.
